# Triazoles and Strobilurin Mixture Affects Soil Microbial Community and Incidences of Wheat Diseases

**DOI:** 10.3390/plants12030660

**Published:** 2023-02-02

**Authors:** Anastasia V. Vasilchenko, Darya V. Poshvina, Mikhail V. Semenov, Vyacheslav N. Timofeev, Alexandr V. Iashnikov, Artyom A. Stepanov, Arina N. Pervushina, Alexey S. Vasilchenko

**Affiliations:** 1 Laboratory of Antimicrobial Resistance, Institute of Environmental and Agricultural Biology (X-BIO), Tyumen State University, 625003 Tyumen, Russia; 2 Laboratory of Soil Carbon and Microbial Ecology, Dokuchaev Soil Science Institute, 119017 Moscow, Russia; 3 Scientific Research Institute of Agriculture for Northern Trans-Ural Region—Branch of Tyumen Scientific Centre SB RAS, 625003 Tyumen, Russia; 4 International Integrated Research Laboratory for the Study of Climate Change, Land Use and Biodiversity, Institute of Environmental and Agricultural Biology (X-BIO), Tyumen State University, 625003 Tyumen, Russia

**Keywords:** non-targeted action, soil microbiome, pesticide contamination, difenoconazole, epoxiconazole, kresoxim-methyl, soil quality

## Abstract

Pesticides are widely used in agriculture as a pest control strategy. Despite the benefits of pesticides on crop yields, the persistence of chemical residues in soil has an unintended impact on non-targeted microorganisms. In the present study, we evaluated the potential adverse effects of a mixture of fungicides (difenoconazole, epoxiconazole, and kresoxim-methyl) on soil fungal and bacterial communities, as well as the manifestation of wheat diseases. In the fungicide-treated soil, the Shannon indices of both fungal and bacterial communities decreased, whereas the Chao1 indices did not differ compared to the control soil. Among bacterial taxa, the relative abundances of *Arthrobacter* and *Sphingomonas* increased in fungicide-treated soil due to their ability to utilize fungicides and other toxic compounds. *Rhizopus* and plant-beneficial *Chaetomium* were the dominant fungal genera, with their prevalence increasing by 2–4 times in the fungicide-treated soil. The genus *Fusarium*, which includes phytopathogenic species, which are notably responsible for root rot, was the most abundant taxon in each of the two conditions but its relative abundance was two times lower in fungicide-treated soils, consistent with a lower level of disease incidence in plants. The prediction of metabolic pathways revealed that the soil bacterial community had a high potential for degrading various pollutants, and the soil fungal community was in a state of recovery after the application of quinone outside inhibitor (Q_o_I) fungicides. Fungicide-treated soil was characterized by an increase in soil microbial carbon, compared with the control soil. Collectively, the obtained results suggest that the application of difenoconazole, epoxiconazole, and kresoxim-methyl is an effective approach for pest control that does not pose a hazard for the soil ecosystem in the short term. However, it is necessary to carry out additional sampling to take into account the spatio-temporal impact of this fungicide mixture on the functional properties of the soil.

## 1. Introduction

Every year, up to 2.5 million tons of pesticides are released into the environment, and then pesticide residues are found in soils. Only a very small proportion of pesticides reach target organisms [1], whereas the rest may pose threats to beneficial microorganisms and the overall ecosystem. Pesticide exposure in soil can potentially have negative effects on soil microbial communities and microbial processes and consequently lead to reduced soil quality [2]. However, the final effect on the soil microbiome after the application of pesticides may be positive, negative, or neutral, depending on the soil’s properties and the chemical nature of the pesticides.

Negative effects of herbicides have been demonstrated for *Rhizobium*, *Chlamydomonas*, *Azotobacter*, *Azospirillum*, and heterotrophic S-oxidizing and S-reducing bacteria [3,4]. At the same time, insecticides, such as carbofuran, carbosulfan, thiomethoxam, imidacloprid, and chlorpyrifos, did not significantly affect the abundance of *Rhizobium* or phosphorus solubilizing bacteria [5]. Despite the fact that fungicides contribute to more than 35% of the global pesticide market [6], their impact on ecosystems has received less attention compared to herbicides and insecticides. Triazoles and strobilurins account for the largest group of commercially available fungicides.

Triazoles were introduced into practice in the 1970s and since then, their market share has been increasing annually. Triazole fungicides disrupt ergosterol biosynthesis in fungal cells by inhibiting the enzyme lanosterol 14α-demethylase, but they do not prevent spore germination or early germ-tube growth [7]. Strobilurins were introduced into the German market in 1996 [8]. Strobilurins are quinone outside inhibitor (Q_o_I) fungicides that bind to the quinol oxidation (Q_o_) site of cytochrome b and inhibit mitochondrial respiration [9].

Although triazoles and strobilurins are known to be effective for major groups of plant pathogenic fungi, little is known about their effects on bacteria. Bacteria do not have sterols or mitochondria, so the usage of triazoles and quinone outside inhibitor (Q_o_I) fungicides is assumed to have no effect on them. However, it was found that triadimefon (triazole) had long-term inhibiting effects on soil bacterial communities [10]. Triticonazole and tebuconazole increased the number of soil bacteria and stimulated dehydrogenase activity [11], whereas two other sterol-targeting fungicides (fenpropimorph and propiconazole) inhibited overall bacterial activity [12]. Metagenomic analysis showed that some soil microbial genera belonging to Proteobacteria and Firmicutes decreased significantly, whereas the abundance of Actinobacteria increased when strobilurins were present in the soil [13]. Often, pesticides and their decomposition products serve as carbon sources for soil microorganisms. However, there are insufficient data on the predominance of pesticide-degrading microorganisms that affect soil quality.

Another problem accompanying the use of fungicides is the emergence of resistant pathogens [14]. Agricultural practices based on using combined fungicides can minimize the emergence of resistant pathogenic clones. Mixing or rotating fungicides with different modes of action is considered to be an effective approach to slow down the development of field resistance [15]. Strobilurin and triazole fungicides have a wide spectrum of activity and their combination significantly reduces the resistance of phytopathogens. Although mixtures of strobilurins and triazoles have been widely studied as plant disease control chemicals, no studies have been conducted on the effect of such a combination on the soil ecosystem [16,17,18]. There are studies that have separately shown the effect of triazoles and strobilurins on the soil’s functional properties and microbiome [1,19].

In this study, we used a fungicide mixture which combined strobilurin kresoxim-methyl and two triazoles (difenoconazole and epoxiconazole) to assess the post-treatment effects of the fungicides’ mixture on the soil fungal and bacterial composition and diversity, as well as on wheat disease incidences. The following questions were addressed: how will the treatment change the microbial activity of the soil? Does a fungicide combining strobilurin with triazoles affect the bacterial community? How has the diversity and the abundance of pathogenic bacterial and fungal taxa changed, and is there a relationship with incidences of diseases in wheat?

## 2. Results

### 2.1. Soil Chemical and Microbiological Properties

The fungicide-treated soil was characterized by a higher microbial biomass carbon (185.10 ± 21.6) compared to the control soil (147.84 ± 13.48) (*p* < 0.001) and a higher carbon-to-nitrogen ratio (*p* < 0.05) (Table 1). The ratio of microbial biomass carbon to soil organic carbon (MBC/SOC) was higher in the fungicide-treated soil (*p* < 0.001) compared to the control soil. The values of basal respiration (BR) and the сoefficient of microbial respiration (QR), the metabolic coefficient (*q*CO_2_), and the *q*CO_2_/SOC ratio, as well as the soil organic carbon (SOC) and total nitrogen (TN), did not differ significantly between the compared experimental groups (Table 1).

Results are presented as mean ± standard deviation (3 biological replicates with 5 analytical replicates each).

### 2.2. The Shift of Bacterial Taxa under Fungicide Treatment

A total of 77,480 reads were obtained for the control soil samples and 24,308–28,588 sequences per sample (mean 25,826 ± 2430) were clustered to 262 ± 6 ASVs. Sequencing of fungicide-treated soil samples revealed 90,893 reads in total, and 23,785–34,150 reads per sample (mean 30,297 ± 5671) were clustered to 256 ± 8 ASVs.

**Figure 1:**
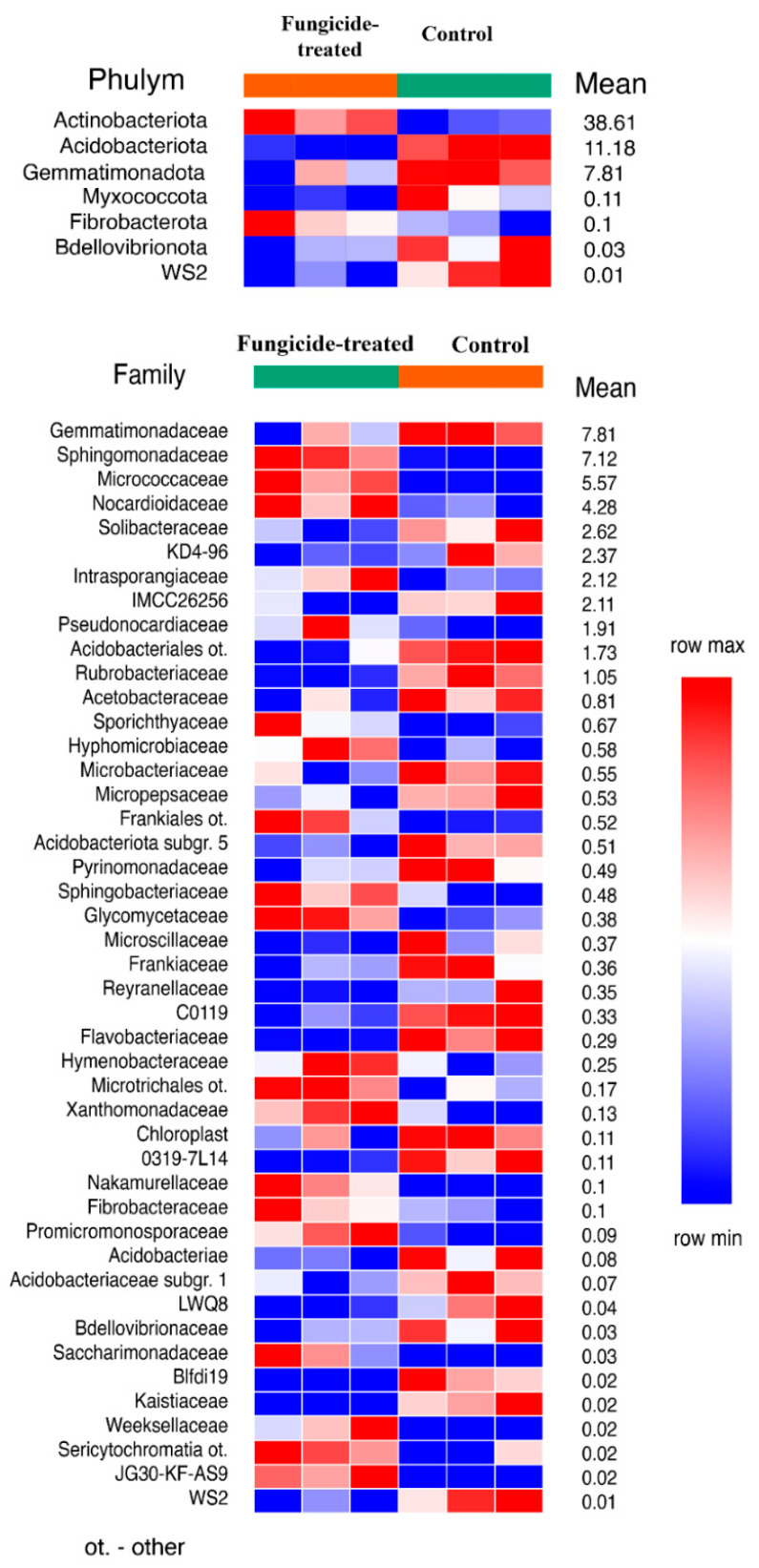
The relative abundance of bacterial phyla and families ranked by their relative abundance. The data represent means of three replicate sequencing reactions, and each reaction was based on pooled DNA samples from three biological replicates (*p* < 0.05).

The estimation of the relative abundance of the dominant bacterial taxa in the studied soils revealed a remarkable shift in bacterial communities under the application of the fungicide (Figure S1). The most abundant bacterial phyla in both soil groups were Actinobacteriota, Proteobacteria, and Acidobacteria (Figure S2). The application of the combined fungicide resulted in an increase in the relative abundance of Actinobacteriota and Fibrobacterota, and a decrease in that of Acidobacteriota, Myxococcota, Bdellovibrionota, Gemmatimonadota, and the WS2 candidate phylum compared to the control soil (*p* < 0.05) (Figure 1). We found that, at the family level, the fungicide-treated soil consisted of 25 families of which the relative abundance was significantly decreased, and 20 families of which the relative abundance was significantly increased compared to the control soil (*p* < 0.05) (Figure 1).

**Figure 2:**
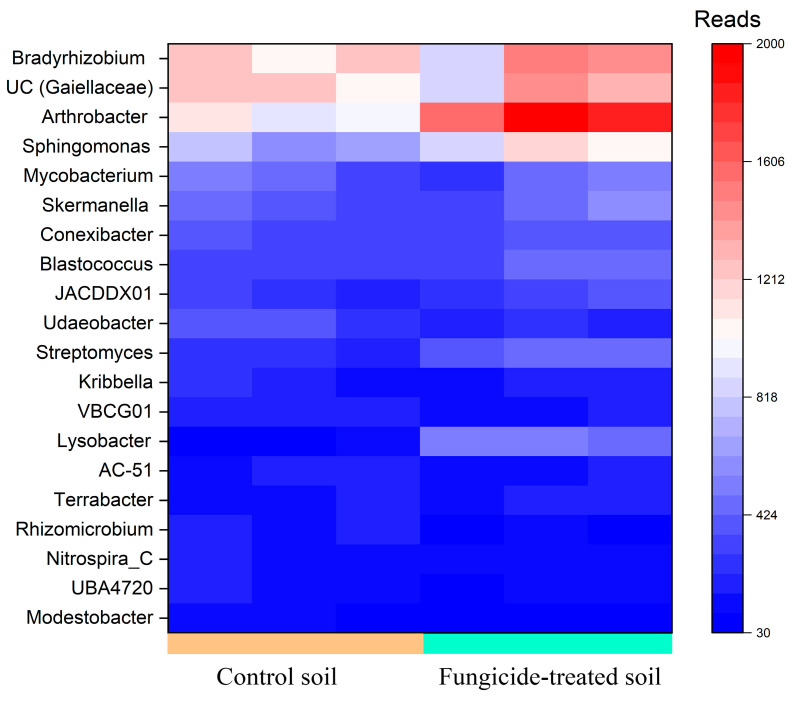
Heatmap of the relative abundance of bacterial genera, estimated on the basis of ASV reads.

Five genera, including *Arthrobacter* and *Bradyrhizobium*, and the unidentified bacteria belonging to *Gaiellaceae*, constituted a major group (>1000 reads, denoted as dominant taxa) in the bacterial community of the control soil. Among these, the relative abundance of *Arthrobacter* (+0.85 ± 0.2 Log2Fold, *p* < 0.01) was significantly increased in the fungicide-treated soil compared to the control soil (Figure 2).

Genera in moderate abundance (500–1000 reads, denoted as moderately abundant taxa) were composed of eight genera in the control soil; however, only two ASVs were identified with the genus *Sphingomonas*. The relative abundances of these *Sphingomonas* spp. significantly increased (+0.64 ± 0.3 and 0.67 ± 0.3 Log2Fold, *p* < 0.05) in the soil treated with the fungicide compared to the control soil (Figure 2).

Among genera that were denoted as low-abundance taxa (100–500 reads), the relative abundances of *Lysobacter* (+2.3 ± 0.3 Log2Fold, *p* < 0.01), *Streptomyces* (+0.86 ± 0.2 Log2Fold, *p* < 0.01), *Modestobacter* (−1.13 ± 0.8 Log2Fold, *p* < 0.05), and *Udaeobacter* (−0.65 ± 0.3 Log2Fold, *p* < 0.01) were significantly altered in the fungicide-treated soil compared to the control soil (Figure 2). Twelve bacterial genera were found to be unique to each of the studied soils (Table S1); however, most of them belonged to unclassified taxa.

### 2.3. Alpha Diversity of the Bacterial Community

**Figure 3:**
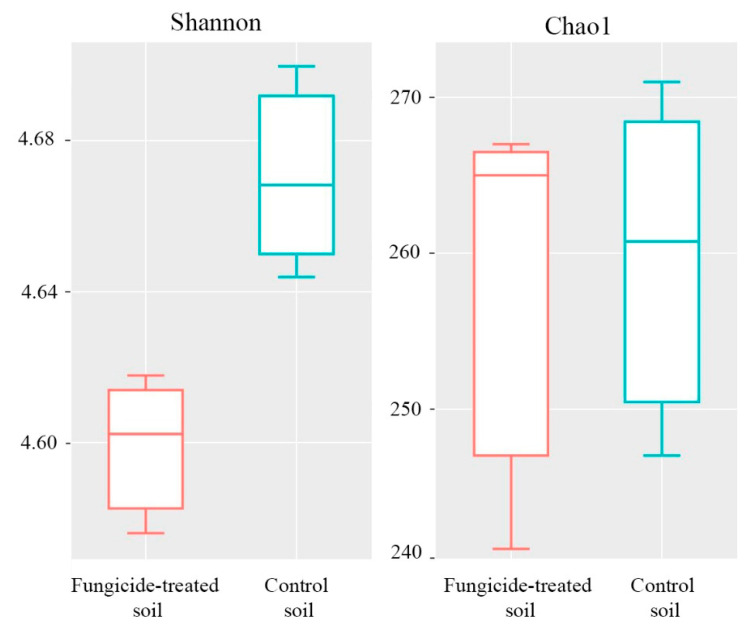
Box plots of the Shannon and Chao1 alpha-diversity indices of bacterial communities for the fungicide-treated and the control soils. The data represent means of three replicate sequencing reactions. Statistical analysis was performed using the Mann–Whitney test.

The Shannon index of bacterial diversity in the fungicide-treated soil was significantly lower than in the untreated soil (*p* < 0.05) (Figure 3). A similar trend was also observed in Chao1 index, although the difference between the two conditions was not significant (*p* > 0.05) (Figure 3).

### 2.4. Functional Potential of the Bacterial Community

**Figure 4:**
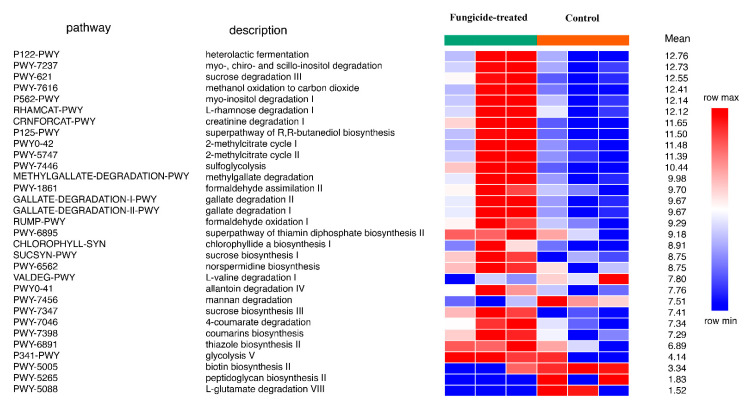
Differentially represented pathways of bacterial community between the control and the fungicide-treated soils. The three columns represent three replicate sequencing reactions, and each reaction was based on pooled DNA samples from three biological replicates (*p* < 0.05).

The reconstruction of metabolic pathways revealed 393 MetaCyc pathways, wherein 31 were differentially represented between the control and the fungicide-treated soils (Figure 4). Among them, 12 pathways were associated with the biosynthesis of vitamins, carbohydrates, and amino acids, whereas 17 pathways were involved in degradation and assimilation processes.

In general, the fungicide treatment led to an increase in the relative abundance of pathways, including those associated with the processes of degradation of various xenobiotics, for example, gallate and methylgallate degradation and formaldehyde oxidation and assimilation (Figure 4). Only five pathways were found to be significantly (*p* < 0.05) increased in their relative abundances in the control soil compared to the fungicide-treated one. These pathways were mannan degradation, L-valine degradation I, L-glutamate degradation VIII, biotin biosynthesis II, and peptidoglycan biosynthesis II (Figure 4).

### 2.5. Shift of Fungal Taxa under the Fungicide Treatment

A total of 75,913 reads were observed for the control soil samples and 12,055–33,761 reads per sample (mean: 25,304 ± 11,619) were clustered to 117 ± 9 ASVs. Sequencing of fungicide-treated soil samples revealed 82,385 reads in total and 20,546–32,556 reads per sample (mean 27,461 ± 6207) were clustered to 112 ± 4 ASVs.

**Figure 5:**
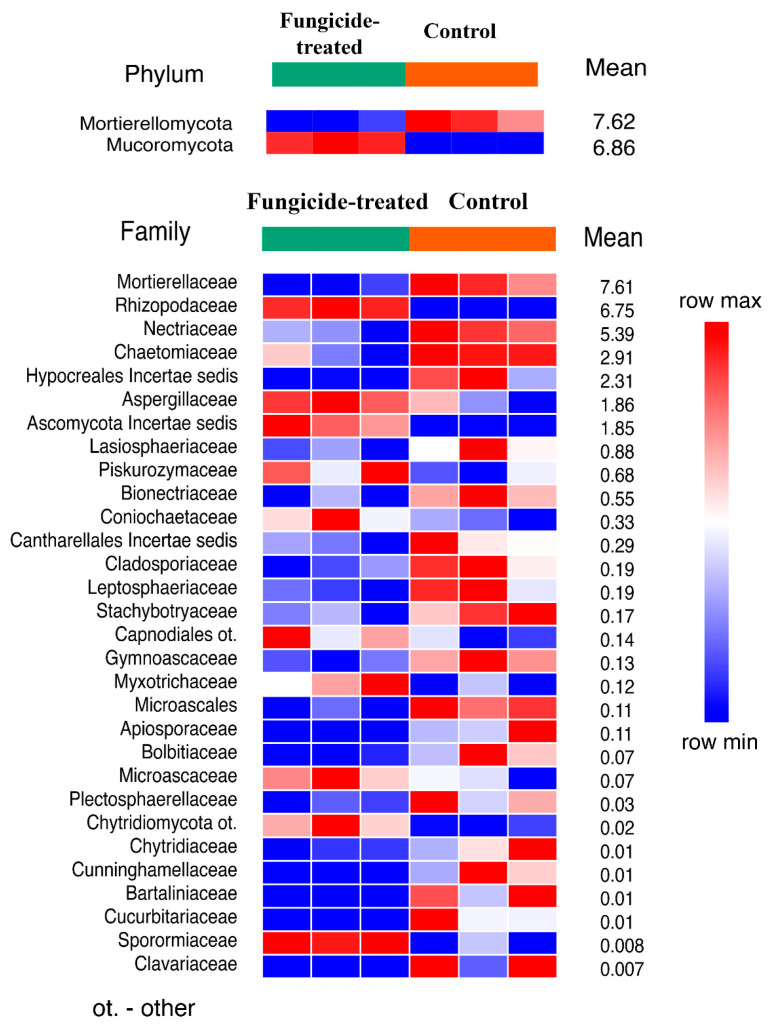
The relative abundance of fungal phyla and families ranked by their relative abundance. The data represent means of three replicate sequencing reactions, and each reaction was based on pooled DNA samples from three biological replicates (*p* < 0.05).

The treatment with fungicide induced a significant shift in soil fungal communities (Figure S1). The fungal community of the control soil was dominated by Ascomycota, Mortierellomycota, and Basidiomycota, whereas in the fungicide-treated soil, the dominant phyla were Ascomycota, Mucoromycota, and Mortierellomycota (Figure S3). Statistical analysis revealed that only Mucoromycota increased and Mortierellomycota decreased (*p* < 0.01) in their relative abundance when the soil was treated with the fungicide (Figure 5). Among the identified families of which the relative abundance significantly changed (30 taxa), most of them (20 families) decreased in terms of their relative abundance under the application of fungicide (Figure 5).

**Figure 6:**
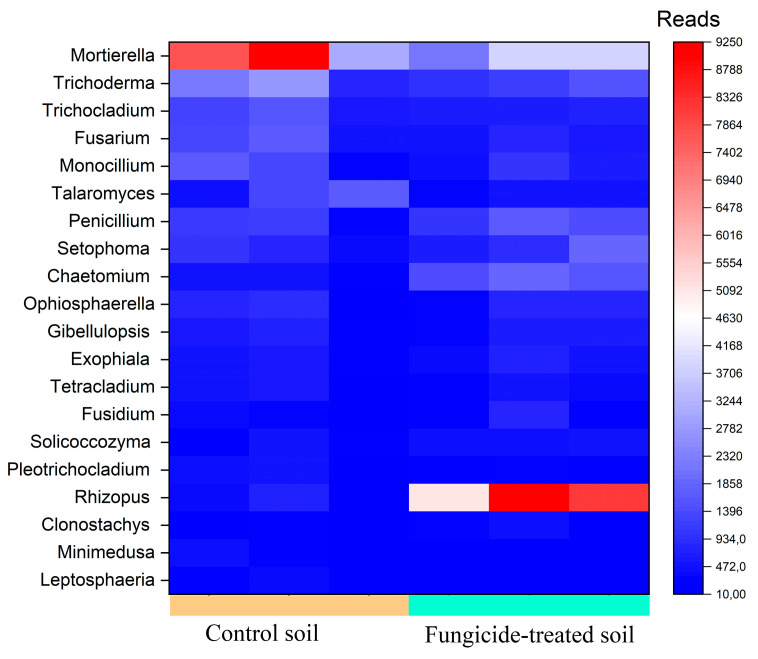
Heatmap of the relative abundance of fungal genera, estimated on the basis of ASV reads.

The most abundant genera (>1000 reads) in the control soil were *Mortierella*, *Trichoderma*, *Trichocladium*, *Fusarium*, *Monocillium*, and *Talaromyces* (Figure 6). However, in the fungicide-treated soil, the relative abundances of *Talaromyces* (−1.24 ± 0.5 Log2Fold, *p* > 0.05), *Mortierella* (−0.93 ± 1.1 Log2Fold, *p* > 0.05), *Trichocladium* (−0.60 ± 0.8 Log2Fold, *p* > 0.05), *Trichoderma* (−0.50 ±1.2 Log2Fold, *p* > 0.05), and *Monocillium* (−0.33 ± 1.6 Log2Fold, *p* > 0.05) were insignificantly reduced compared to the control soil (Figure 6). Among the dominant taxa of the control soil, the phytopathogenic *Fusarium* genus was found, whereas in the fungicide-treated soil, its relative abundance was insignificantly reduced (−0.76 ± 0.9 Log2Fold, *p* > 0.05).

We recovered five fungal genera with a moderate abundance (500–1000 reads) (Figure 6). Among these genera, *Penicilium* showed the highest Log2Fold change (+0.88 ± 1.3 Log2Fold, *p* > 0.05), whereas the relative abundance of *Setophoma* (+0.63 ± 1.6 Log2Fold, *p* > 0.05), *Ophiosphaerella* (+0.55 ± 2.4 Log2Fold, *p* > 0.05), *Gibellulopsis* (+0.24 ± 1.4 Log2Fold, *p* > 0.05), and *Fusarium* (−0.19 ± 1.3 Log2Fold, *p* > 0.05) did not vary significantly between the two conditions.

The most pronounced shift occurred with genera with a low abundance (denoted as minor taxa) (100–500 reads). The highest increase in relative abundance was observed for *Rhizopus* (+4.5 ± 1.1 Log2Fold, *p* < 0.01) and *Chaetomium* (+2.23 ± 0.8 Log2Fold, *p* < 0.01), whereas the lowest decrease was observed for the genus *Minimedusa* (−1.85 ± 0.9 Log2Fold, *p* > 0.05) (Figure 6).

Several fungal genera containing plant pathogens were recovered with a low abundance, including *Septoriella* (−2.10 ± 1.8 Log2Fold, *p* > 0.05), *Aspergillus* (−1.04 ± 1.3 Log2Fold, *p* > 0.05), *Alternaria* (+0.18 ± 1.9 Log2Fold, *p* > 0.05), and *Bipolaris* (−0.60 ±1.5 Log2Fold, *p* > 0.05). We noticed that six fungal genera were only recovered from control soils, whereas two were only detected in fungicide-treated soils (Table S1).

### 2.6. Alpha Diversity of the Fungal Community

**Figure 7:**
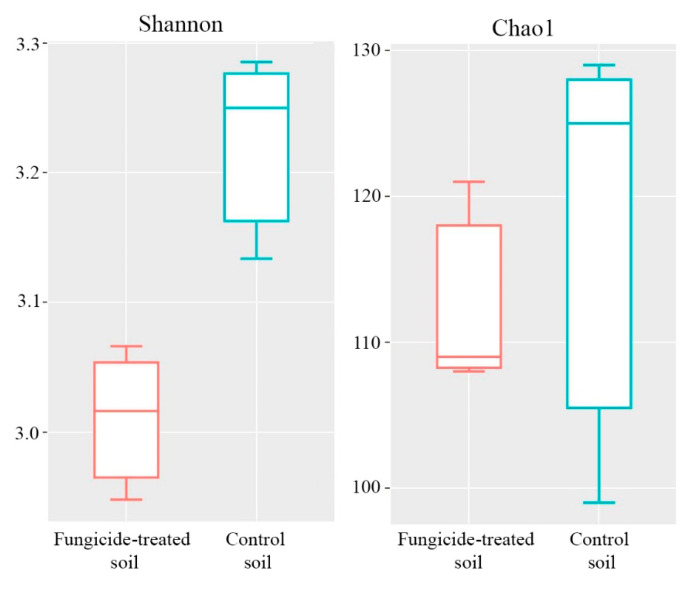
Box plots of the Shannon and Chao1 alpha diversity indices of fungal communities in the fungicide-treated and the control soils. The data represent means of three replicate sequencing reactions. Statistical analysis was performed using the Mann–Whitney test.

The Shannon and Chao1 indices were used to represent the alpha diversity of the community. The fungicide application significantly reduced the Shannon index of the fungal community (*p* < 0.05) (Figure 7), whereas the Chao1 index differed insignificantly between the two treatments (Figure 7).

### 2.7. Functional Potential of the Fungal Community

**Figure 8:**
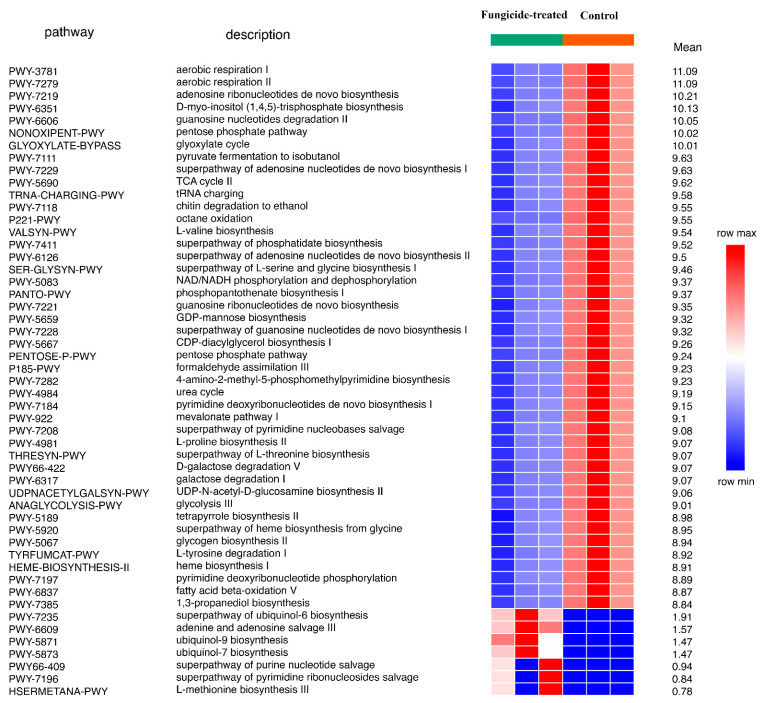
Differentially represented pathways of fungal community between the control and the fungicide-treated soils. The three columns represent three replicate sequencing reactions, and each reaction was based on pooled DNA samples from three biological replicates (*p* < 0.05).

Using the ITS sequencing data, 66 MetaCyc pathways were completely restored. Of these, 51 were differently represented between groups (*p* < 0.05). Only seven pathways, which were related to ubiquinol and nucleic acid biosynthesis, were discovered to be more abundant in the fungicide-treated soil (Figure 8).

### 2.8. Effect of Soil Management on Fungal Diseases

**Figure 9:**
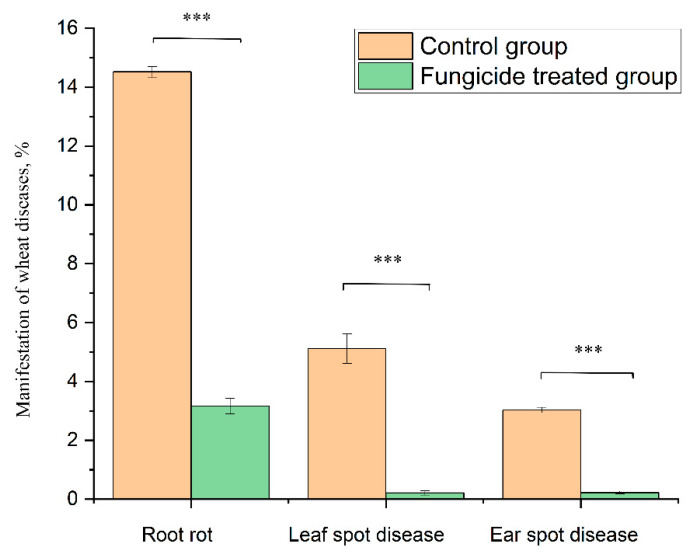
Wheat disease severity and incidence in the control and fungicide-treated plants. Data present mean ± standard deviation (n = 50). *** *p* < 0.001.

At the beginning of the growing season, 8.25% of the plants without fungicide treatment (grown in the control field) showed symptoms of root rot. At the end of growing season, infected plants increased up to 14.52% ± 0.18% (Figure 9). By contrast, fungicide treatment protected 100% of the plants in the tillering phase, and up to 96.8% ± 0.27% at the end of the growing season (Figure 9).

The treatment of wheat with the studied fungicide mixture reduced the manifestation of the leaf spot disease caused by *Zymoseptoria tritici*. The disease severity was 5.11% ± 0.50% in the control group and 0.21% ± 0.07% in the fungicide-treated group (*p* < 0.001). The absence of fungicide in the plant-protection scheme resulted in the appearance of ear spot disease caused by *Stagonospora nodorum*. The disease incidence rate in the control group was 3.03% ± 0.10%, and the use of fungicide reduced this value to 0.22% ± 0.05% (*p* < 0.001) (Figure 9).

## 3. Discussion

We found that the combined use of these fungicides affected both soil bacterial and fungal communities. Bacterial and fungal taxa were clustered into three conditional groups depending on their relative abundances: dominant, moderately abundant, and minor taxa. There were a few features of the microbial response to this treatment. Bacterial and fungal diversity decreased under the influence of the combined fungicide. Moreover, bacterial and fungal communities differed in their responses to the treatment. All dominant taxa in the fungicide-treated soil remained the same as in the control soil, but their relative abundances changed.

In the fungicide-treated soil, the relative abundance of *Arthrobacter* increased. This bacterial genus is known for its ability to decompose strobilurins as a source of carbon [20]. The relative abundance of *Sphingomonas* also increased. Members of *Sphingomonadaceae* are known to have the ability to degrade a variety of aromatic compounds [21]. Many Sphingomonads have been isolated from environments contaminated with pesticides, herbicides, and other xenobiotics. These products can be used by bacteria as a sole carbon source [22]. Recently, two strains of *Sphingomonas* spp. were isolated from wheat grains and demonstrated the ability to degrade propiconazole [23]. Thus, at least three bacterial genera found in the studied soils could degrade strobilurins and triazoles. This may explain the increase in the relative abundance of these genera in the fungicide-treated soil. In the fungicide-treated soil, there was an increase in the relative abundance of *Streptomyces*, which have a high potential as biofertilizers and biocontrol agents due to their ability to produce a wide spectra of bioactive compounds, antibiotics, and extracellular enzymes [24,25]. The most pronounced shifts occurred with minor taxa; some of these taxa were not detected in the fungicide-treated soil, whereas a few were recovered from the fungicide-treated soil that were not found in the control soils. However, most minor taxa belonged to the non-classified uncultivated genera, and their environmental role is unknown.

The fungal community was affected the most by the fungicide mixture. *Rhizopus* was the most relatively abundant taxon in the fungal community, and its relative abundance in the fungicide-treated soil increased significantly. *Rhizopus* species are known as soil saprotrophs and are capable of the sorption of various toxicants [26]. Since the relative abundance of this taxon increased by more than eight times, it is possible that *Rhizopus* spp. may play a more specific ecological role related to fungicide applications. Another fungal taxon of which the relative abundance significantly increased in the fungicide-treated soil was the *Chaetomium* genus, which is known to include species with plant-beneficial traits. For example, several *Chaetomium* species are known as biocontrol agents of various phytopathogenic fungi, such as *Fusarium*, *Helminthosporium*, *Pythium*, *Alternaria*, and *Phytophthora* [27,28,29,30].

The common root rot infection of wheat is facilitated by the presence of pathogens on seeds and in the soil. The intensity of damage to the root system can vary under the influence of weather conditions (precipitation, temperature). Phytopathogens belonging to the *Septoriella*, *Bipolaris*, and *Alternaria* genera were found in the fungicide-treated soil in lower numbers or were even absent compared to the control soil. For example, *Septoriella hirta* is considered an economically important secondary pathogen that increases the cost of harvesting and reduces the quality of the grain [31]. However, the abundances of all of these fungi were low (less than 100 reads). On the contrary, *Fusarium* was found to be the most abundant phytopathogen present in the soil without the fungicide treatment. This fungal genus includes many species that are able to colonize the lower stems (crowns) of wheat (*Triticum aestivum* L. subsp. *aestivum*) and durum wheat (*Triticum turgidum* L. ssp. *durum*), and they can be associated with crown and foot rot, as well as head blight [32,33,34]. Moreover, many species of *Fusarium* genus produce toxins, among which the most common mycotoxin groups are trichothecenes, zearalenones, and fumonisins [35]. In our study, the relative abundance of *Fusarium* decreased two times when the combined fungicide was applied, and it was potentially associated with a 50% reduction in the incidence of root rot.

Qualitative and quantitative shifts in the microbiome are important indicators that determine changes in the functional properties of soils. We used PICRUSt2 software to predict the functional potential of bacterial or fungal communities via taxonomic profiling [36]. We found out that the treatment of plants with studied fungicides mainly impacted fungal-associated biosynthetic pathways. Interestingly, the metabolic pathways prevailing in the fungicide-treated soil were associated with the biosynthesis of ubiquinones—components of the mitochondrial respiratory chain [37]—and with the biosynthesis of purine and pyrimidine bases. A possible explanation for this is the fact that kresoxim-methyl belongs to a fungicide from the class of QoIs (quinone outside inhibitors), which affect cell respiration and ATP formation. Similar results were obtained in a recent study of a soil treated with the fungicide chlorothalonil [38], which inhibits thiol-dependent enzymes and decreases the intracellular level of reduced glutathione involved in respiration [39,40]. The reconstruction of the metabolic pathways of the microbiome in fungicide-treated soil showed an overrepresentation of pathways associated with anaerobic respiration and the degradation of organosulfonate compounds [38].

The prediction of the microbial functional profile also revealed that the relative abundances of metabolic pathways associated with the conversion of various xenobiotics were higher in the fungicide-treated soil. Thus, the functional reactions of the soil community are specific depending on the class of fungicide applied, and these reactions can be determined using in silico methods. However, the description of functional changes in microbial communities under fungicide treatment should be based on a direct assessment of several important microbiological indicators of soil microbial activity.

We found that the predicted microbial biomass increased in the fungicide-treated soil. The application of fungicides led to the death of fungi, which caused the accumulation of necromass. Necromass, as an organic substance, serves as an available source of nutrients for other groups of microorganisms [41,42]. The part of the microbial community that is resistant to fungicide begins to grow in conditions of low competition for this available substrate, which leads to an increase in the measurable value of the microbial biomass [43]. The storage of organic matter in the microbial biomass is also indicated by the high MBC/SOC value observed in the fungicide-treated soil compared to the control soil. The high MBC/SOC ratio implies that more carbon was present in a form that was available for microorganisms [44]. We also found that there was no significant increase in the respiration rate or the *q*CО_2_/SOC value of the microbial community treated with the fungicides. It can be argued that in the treated soil, the efficiency of the utilization of organic matter by soil microorganisms (*q*CО_2_/SOC) and the metabolic efficiency of microbial communities were consistent with those of the control soils. The microbial respiration coefficient (QR) and metabolic coefficient (*q*CО_2_) were used to assess environmental stress in the microbial communities. Lower values of *q*CО_2_ and QR indicated the ‘optimal’ functioning of the microbial community. We found that the decreases in *q*CО_2_ and QR values in fungicide-treated soil were insignificant. The obtained values of microbial respiration (QR) and metabolic coefficients (*q*CО_2_) indicated that the microbial community of the control soil, as well as that of the fungicide-treated soil, were in a stable state.

## 4. Materials and Methods

### 4.1. Site and Sampling

Soils were sampled in a field at the Research Institute of Agriculture of the Northern Trans-Urals (Coordinates: 57.094, 65.376). Geographical zone: subtaiga subzone of the Tavda province (Turin subprovince). The area is a gently sloping plain with pine (*Pinus sylvestris* L.) and birch grassy (*Betula pendula* L.) mixed forests. (*Tríticum durum* Desf.). The soil type under the study was Luvic Phaeozems. Before sowing spring wheat (*Tríticum durum* Desf.), a special agrotechnical process was applied: the first plowing was in autumn of 2019 and then plowing in early spring of 2020, followed cultivation during the summer of 2020. Spring wheat was sowing in May 2021.

The applied fungicide preparation was “Terapevt-Pro”, (Zemlyakoff, Moscow, Russia). “Terapevt-Pro” contains (gram per liter): difenoconazole—80; kresoxim-methyl—125; epoxiconazole—125. The fungicide mixture was applied once during the earing phase of wheat on 7 July 2021 at the rate of 0.7 L per ha. The control (untreated with fungicides) group of soils was located at a distance of 20 m. 

Soils were sampled in September 2021 after wheat harvest. Soil samples were collected using the checkerboard sampling method. Three spatially distant plots (1 m^2^) were randomly laid in each studied area. Samples were taken from 0–5 cm of the upper humus soil layer at four points in the corners and one in the center of the plot. For each plot, a pooled sample was prepared by mixing incremental samples. The maximum time from the moment of sampling to their arrival at the laboratory was no more than 2 h. The soil samples were sieved with a mesh size of 2 mm and stored at the temperature of minus 80 °C. Thus, three bags of soil were collected from each area.

The soil texture was presented by clay (28%), silt (26%) and sand (clay loam) (46%). The acidity of the both studied soils was pH_KCl_ 5.89 and pH_H2O_ 7.06. Soil pH was measured according to the international standard ISO 10390. The pH_KCl_ value was determined potentiometrically in a 1 M KCl solution and the pH_H2O_ value was determined in an aqueous solution at a soil:solution ratio of 1:5 using a pH-meter Orion Star A 111 (Thermo Scientific, Waltham, MA, USA) [45]. The total carbon (TC) and total nitrogen (TN) contents were measured using a Vario EL III elemental analyzer (Elementar, Langenselbold, Germany). The content of total C determined in this way was considered equal to the SOC content, since carbonates were absent in the studied Luvic Phaeozems.

### 4.2. Determination of Soils’ Microbiological Properties

Soil basal respiration (BR, CO_2_-C) was determined based on the rate of the CO_2_ release from the soil during 24 h of incubation at 22 °C and 60% of the full moisture capacity using a Trace GC Ultra gas chromatograph with a DSQ II mass-selective detector (Thermo Electron Corporation, Waltham, MA, USA). BR was expressed in μg CO_2_-C g^−1^ soil h^−1^. The soil microbial biomass was determined by means of the substrate-induced respiration method (SIR) [46]. A soil sample (5 g) was placed in a 100 mL bottle and a glucose solution (0.1 mL/g soil, 10 mg/g soil) was added. The vial was sealed and the time of the beginning of incubation with the substrate was recorded. After 3–5 h of incubation, an air sample was taken from the vial and analyzed using a gas chromatograph. The exposure time was strictly fixed. The SIR rate was expressed in μg CO_2_-C g^−1^ soil h^−1^ [47,48]. The MBC values were expressed in μg C g^−1^ soil. Basal soil respiration and microbial biomass were determined for three biological replicates (five analytical replicates each).

The coefficient of microbial respiration (QR) was calculated on the basis of the relationship between the basal and substrate-induced respiration: QR = BR/SIR [49]; the metabolic coefficient or specific respiration of microbial biomass was calculated as *q*CО_2_ = BR/MBC, μg CO_2_-C mg^−1^ MBC h^−1^ [50].

### 4.3. Phytosanitary Control of Spring Wheat

The development of common root rot was determined by means of visual inspections, which were performed twice during the growing season in the tillering phase and before harvesting the crop [51]. Symptoms of root rot included poor emergence and root development, yellowing, and discolored roots, as well as various lesions on root or stem tissue near the soil.

The development of aerogenic infections (foliar and blotch disease of wheat) was monitored from the tillering phase to milky ripeness. Plant material for analysis was taken from a wheat field at three (root rot) and five (aerogenic infections) spatially distant points. The degree of damage to the leaf surface caused by *Zymoseptoria tritici* (a foliar disease of wheat) or damage to the wheat ear caused by *Septoria nodorum* was expressed as a percentage using the universal scale [52].

### 4.4. Total Soil DNA Isolation and Sequencing

Total soil DNA was isolated from 0.5 g of soil using the FastDNA™ Spin Kit for Soil DNA Extraction (MP Biomedicals, Moscow Russia), according to the manufacturer’s protocol. DNA was extracted from three technical replicates per sample to minimize the DNA extraction bias (9 samples of DNA were obtained in total). To assess the yield of total soil DNA, the absorbance was measured at 230, 260, and 280 nm using a NanoPhotometer N120 (Implen, Westlake Village, CA, USA). The quality of total soil DNA was estimated using the following absorbance ratios: A260 nm/230 nm (DNA/humic acid) and A260 nm/280 nm (DNA/protein). DNA yield was also quantified fluorometrically with a Qubit 4.0 fluorometer (Thermo Fisher Scientific, Waltham, MA, USA). Both a NanoPhotometer N120 and the Qubit instrument were used according to the manufacturers’ protocols. The extracted DNA was stored in a freezer (−80 °C) until further analysis.

The 16S rRNA sequencing libraries were constructed according to the Illumina 16S Metagenomic Sequencing Library Preparation protocol (Illumina) targeting the V3, V4 hypervariable regions of the 16S rRNA genes using primers S-D-Bact-0341-b-S-17 (5′-CCTACGGGNGGCWGCAG-3′) and S-D-Bact-0785-a-A-21 (5′-GACTACHVGGGTATCTAATCC-3′) [53]. The “xGen™ ITS1 Amplicon Panel” was used for fungal community profiling following the manufacturer’s instructions (Integrated DNA technologies, Inc., Redwood City, CA, USA). The amplicon sequencing was carried out using MiSeq Illumina sequencer (Ilumina Inc., San Diego, CA, USA) and a set of paired ends v2 Illumina (cluster generation and paired ends sequencing with the power of 2 × 250 bp).

### 4.5. Data Analysis and Statistics

The quality of sequenced data was estimated using FastQC v 0.11.9 software [54]. The reads were filtered and trimmed using the Trimmomatic v0.36 program. Reads were removed when the average quality throughout the entire length was at least 31 (both for forward and reverse reads). After filtering, the data were checked using the FastQC program.

The reads were further processed using the DADA2 v4.0.3 package [55] of the R language. Using DADA2, we performed additional filtering of sequences by quality and the pruning of reads based on the following quality parameters: reads less than 232 bp in length were filtered out. To identify “unique” reads, counting the number of repetitions of these sequences among the analyzed data, sequence dereplication was carried out. Recovery of the amplicon sequence variant (ASV) was performed using the main function of the dada2 algorithm. During the preprocessing of amplicon information using the dada2 package, chimeric sequences were removed. The normalization of ASVs was carried out using the “phyloseq_to_deseq2” function in the Deseq2 package. lfcShrink (shrinkage estimation of logarithmic fold changes) normalization was applied to the Deseq object.

The phylogenetic composition of bacterial communities was determined by comparing the V3-V4 16S rRNA nucleotide sequences with the reference rRNA sequences from the GTDB database using the IdTaxa function in the DECIPHER package [56]. The phylogenetic composition of fungal communities was determined by comparing the ITS1 nucleotide sequences with the reference sequences from the UNITE v 8.3 database 10 May 2021.

Alpha-diversity analysis was carried out using the “estimate_richness” function in the phyloseq v3.14 package [57], and the Chao1 and Shannon indices were calculated. The nonparametric Mann–Whitney test was used to determine the influence of the fungicide treatment on the alpha diversity. The effect was considered significant at *p* < 0.05. The alpha-diversity indices were visualized using the box-and-whisker chart function from the ggplot2 v3.3.5 package [58]. The dissimilarity in the community compositional structure between groups (the control soil and the fungicide-treated soil) was assessed using the Bray–Curtis dissimilarity coefficient. The differences in the relative abundance between soil groups were visualized using principal component analysis (PCA) and with heatmaps using the Limma package in the R language [59]. Differences were considered significant at *p* < 0.05.

The measured soil microbiological and chemical properties were statistically manipulated using Origin 2021 (OriginLab Corporation, Northampton, MA, USA). The Shapiro–Wilk test was used to assess the normal distribution of values. In the presence of a normal distribution, the two-sample *t*-test was used, whereas if normality was rejected, the Mann–Whitney test was used. Differences were considered significant at *p*-values < 0.05.

### 4.6. Analysis of Soil Functional Potential

Using the 16S rRNA or ITS-based ASV tables and the reference sequences generated by QIIME2, the functional potential of the microbial communities was predicted using PICRUSt2 software (v2.3.0) [36]. The values were converted using the logarithmic transformation Log2. The data were visualized and the statistical indicators were calculated using the Phantasus web application (v1.11.0).

## 5. Conclusions

We showed that the difenoconazole–kresoxim-methyl–epoxiconazole mixture provided a sufficient level of protection against phytopathogens in wheat. Moreover, this combined fungicide had a restructuring effect on the soil microbiome. The decrease in the abundance of phytopathogenic fungal taxa observed in the soil could potentially reduce the probability of disease infection in subsequent years. At the same time, the studied fungicide preparation had an effect on non-targeted bacteria, probably through changes in the ecological relationships between microorganisms. Our analysis of the structure of the soil microbiome and its biosynthetic potential revealed signs of the restoration of the ecological characteristics of the fungicide-treated soil. These structural changes were reflected in measurable soil health characteristics such as soil microbial carbon content.

Overall, the data obtained in this study indicate that combined fungicides based on strobilurins and triazoles provide a high level of plant protection with an acceptable impact on the soil system, but long-term studies are needed to assess the time frame for achieving functional and structural stability of the microbial community.

## Figures and Tables

**Table 1:** Soil chemical and microbiological properties.

Parameters	Research Sites		*p*-Value
	Сontrol Soil	Fungicide-Treated Soil	
SOС, (g kg^−1^)	19.62 ± 6.85	15.10 ± 4.32	0.27 ^a^
TN, (g kg^−1^)	2.52 ± 1.04	1.69 ± 0.83	0.066 ^b^
C:N ratio	8.23	10.19	0.040 ^b^
BR, μg CO_2_-C g^−1^ soil h^−1^	0.44 ± 0.04	0.58 ± 0.09	0.07 ^a^
MBC, μg C g^−1^ soil	147.84 ± 13.48	185.10 ± 21.6	0.0001 ^a^
QR	0.14 ± 0.02	0.13 ± 0.04	0.59 ^a^
*q*CО_2_, μg CO_2_-C mg^−1^ MBC h^−1^	3.34 ± 0.53	3.14 ± 0.96	0.59 ^a^
MBC/SOC, %	0.73 ± 0.14	1.23 ± 0.14	0.0001 ^a^
*q*CO_2_/SOC, μg CO_2_-C mg^−1^ MBC h^−1^ (g SOC g^−1^ soil)^−1^	1.69 ± 0.27	2.08 ± 0.64	0.08 ^a^

^a^ Mann–Whitney test; ^b^ Two-sample *t*-test; SOС—soil organic carbon; TN—total nitrogen; BR—basal respiration; MBC—microbial biomass carbon; QR—coefficient of microbial respiration; *q*CО_2_—metabolic coefficient. MBС/SOC—share of microbial biomass carbon in organic carbon. *q*CO_2_/SOС—relationship between C-use efficiency and the available soil organic carbon soil.

## Data Availability

The raw sequencing reads were deposited in the NCBI database with the BioProject number PRJNA922337 (accessed on 20 January 2023 https://www.ncbi.nlm.nih.gov/sra/PRJNA922337).

## Supplementary Materials

The following supporting information can be downloaded at: https://www.mdpi.com/article/10.3390/plants12030660/s1. Figure S1: Principal Component Analysis (PCoA) plots of the Bray–Curtis distances between bacterial (a) and fungal (b) families in experimental conditions (the fungicide-treated samples versus the control samples); Figure S2: Average relative abundance of bacterial phyla (a) and families (b) in the studied soil samples; Figure S3: Average relative abundance of fungal phyla (a) and families (b) in the studied soil samples. Table S1: The genera of soil bacteria and fungi that were not detected in 16S rRNA and ITS sequence libraries.
