# Supplementary material for: Triazoles and Strobilurin Mixture Affects Soil Microbial Community and Incidences of Wheat Diseases
Source: Plants (Basel). 2023 Feb 2;12(3):660. doi: 10.3390/plants12030660 (PMC9919142; doi:10.3390/plants12030660)
Supplement: Supplementary file 1 [file plants-12-00660-s001.zip › plants-1938996-supplementary.pdf]

Supplementary material

Triazoles and Strobilurin Mixture Affects Soil Microbial Community and Incidences of Wheat Diseases

Anastasia V. Vasilchenko [1,†], Darya V. Poshvina [1,†], Mikhail V. Semenov [1,2], Vyacheslav N. Timofeev [3], Alexandr V. Iashnikov [1], Artyom A. Stepanov [1], Arina N. Pervushina [4] and Alexey S. Vasilchenko [1,*]

[1] Laboratory of Antimicrobial Resistance, Institute of Environmental and Agricultural Biology (X-BIO), Tyumen State University,625003, Tyumen, Russia
[2] Laboratory of Soil Carbon and Microbial Ecology, Dokuchaev Soil Science Institute, 119017, Moscow, Russia
[3] Scientific Research Institute of Agriculture for Northern Trans-Ural Region—Branch of Tyumen Scientific Centre SB RAS,625003, Tyumen, Russia
[4] International Integrated Research Laboratory for the Study of Climate Change, Land Use and Biodiversity, Institute of Environmental and Agricultural Biology (X-BIO), Tyumen State University,625003, Tyumen, Russia

* Correspondence: avasilchenko@gmail.com
# AVV and DVP contributed equally to this paper

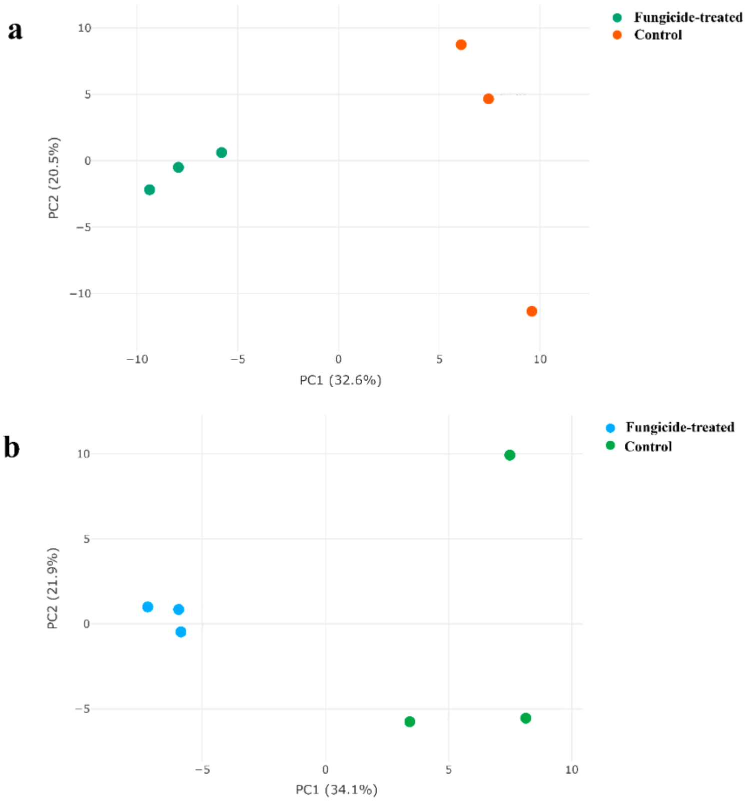

Figure S1. Principal Component Analysis (PCoA) plots of the Bray–Curtis distances between bacterial (a) and fungal (b) families in experimental conditions (the fungicide-treated samples versus the control samples).

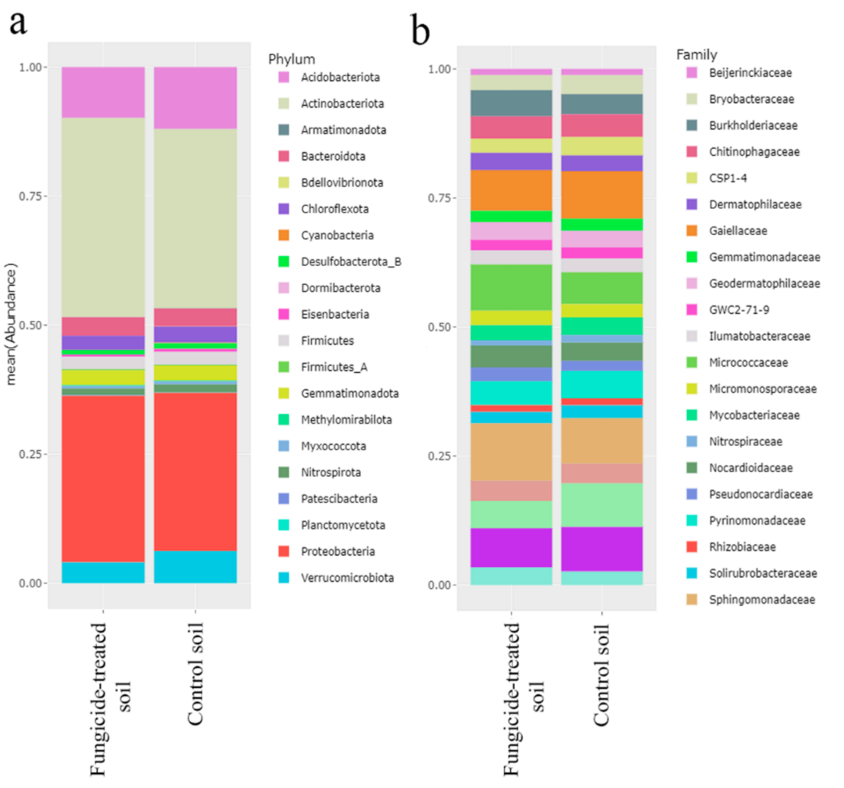

Figure S2. Average relative abundance of bacterial phyla (a) and families (b) in the studied soil samples.

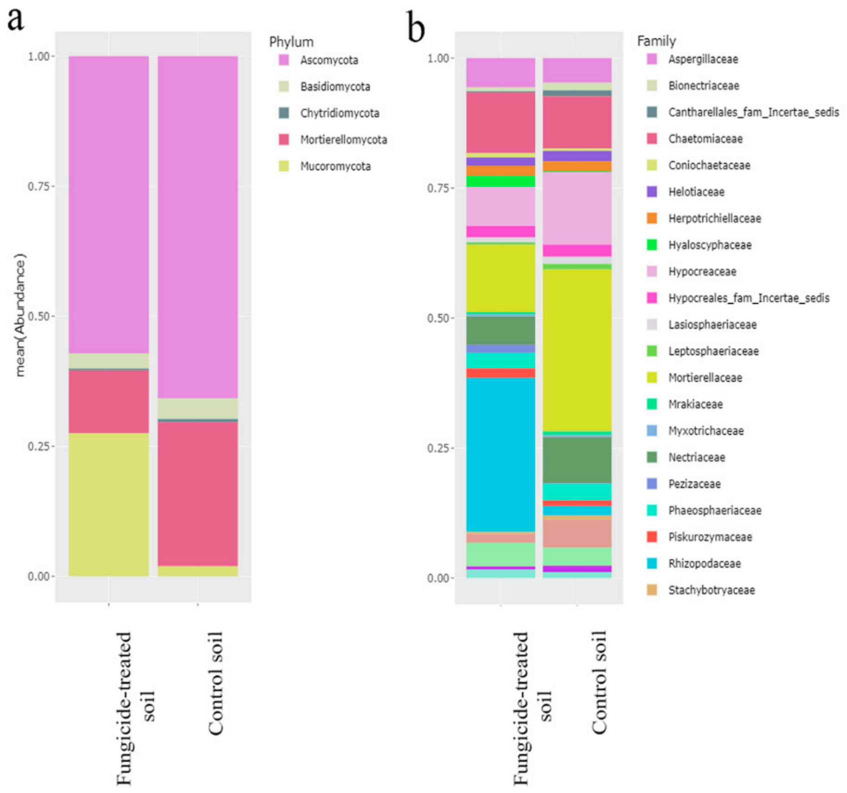

Figure S3. Average relative abundance of fungal phyla (a) and families (b) in the studied soil samples.

Table S1. The genera of soil bacteria and fungi that were not detected in 16S rRNA and ITS sequence libraries

**Bacteria which are absent in the fungicide treated soil**	**Bacteria which are absent in the intact soil**	**Fungi which are absent in the fungicide treated soil**	**Fungi which are absent in the intact soil**
Tardiphaga	*Glycomyces*	*Schizothecium*	*Arthrographis*
Chryseolinea	*Actinosynnema*	*Gliomastix*	*Lecythophora*
Salinisphaera_A	*Hyphomicrobium*	*Idriella*	
Devosia	*Promicromonospora*	*Agrocybe*	
Kaistia	*Pseudoduganella*	*Conocybe*	
Luteitalea	*Chryseobacterium*		
UBA1315	QHVH01		
Flavobacterium	DP-6		
PMG-095	17J80-11		
Herminiimonas	Ga0077541		
Opi-474	JACDCK01		
SHYK01	Palsa-1392		
